# Inhibition of BCL9 Modulates the Cellular Landscape of Tumor-Associated Macrophages in the Tumor Immune Microenvironment of Colorectal Cancer

**DOI:** 10.3389/fphar.2021.713331

**Published:** 2021-09-10

**Authors:** Zhuang Wei, Mengxuan Yang, Mei Feng, Zhongen Wu, Rina Rosin-Arbesfeld, Jibin Dong, Di Zhu

**Affiliations:** ^1^ Department of Pharmacology, School of Basic Medical Sciences, Fudan University, Shanghai, China; ^2^ Key Laboratory of Systems Biology, Innovation Center for Cell Signaling Network, CAS Center for Excellence in Molecular Cell Science, Institute of Biochemistry and Cell Biology, Shanghai Institutes for Biological Sciences, Chinese Academy of Sciences, Shanghai, China; ^3^ Minhang Branch, Zhongshan Hospital, Fudan University, Shanghai, China; ^4^ Department of Pharmacology, School of Pharmacy, Fudan University, Shanghai, China; ^5^ Department of Microbiology and Immunology, Sackler Faculty of Medicine, Tel Aviv University, Tel Aviv, Israel; ^6^ Key Laboratory of Smart Drug Delivery, State Key Laboratory of Molecular Engineering of Polymers, School of Pharmacy, Ministry of Education, Fudan University, Shanghai, China; ^7^ Shanghai Engineering Research Center of ImmunoTherapeutics, Fudan University, Shanghai, China

**Keywords:** colorectal cancer, tumor-associated macrophages, wnt signaling, BCL9, tumor immune microenvironment

## Abstract

Tumor-associated macrophages (TAMs) are an indispensable part of the tumor microenvironment (TME), and they likely play a negative rather than positive role in cancer treatment. However, the cellular landscape and transcriptional profile regulation of TAMs in the case of tumor gene inactivation or chemical interference remains unclear. The B-cell lymphoma 9/B-cell lymphoma 9-like (BCL9/BCL9L) is a critical transcription co-factor of β-catenin. Suppression of Bcl9 inhibits tumor growth in mouse models of colorectal cancer (CRC). Here, we studied the TAMs of CRC by single-cell sequencing. Bcl9 depletion caused macrophage polarization inhibition from M0 to M2 and changed the CRC TME, which further interferes with the inflammation of M0 and M1. The transcription factor regulating these processes may be related to the Wnt signaling pathway from multiple levels. Furthermore, we also found that the cells delineated from monocyte to NK-like non-functioning cells were significantly different in the BCL9-deprived population. Combining these data, we proposed a TAM-to-NK score to evaluate the dynamic balance in TME of monocyte/TAM cells and NK-like non-functioning cells in The Cancer Genome Atlas (TCGA) clinical samples to verify the clinical significance. We demonstrated that the cell type balance and transcription differences of TAMs regulated by BCL9-driven Wnt signaling affected immune surveillance and inflammation of cancer, ultimately affecting patients’ prognosis. We thereby highlighted the potential of targeting Wnt signaling pathway through cancer immunotherapy.

## Introduction

Tumor-associated macrophages (TAMs) are the most significant cluster of immune cells infiltrating colon cancer (Li et al., 2020). TAMs play a central role in the formation of the tumor microenvironment (TME) by secretion of cytokines and chemokines. The roles and phenotypes of TAMs in colorectal cancer (CRC) are controversial, however, and previous studies have reported opposite results. Inhibition of CRC cell growth was achieved with co-culturing CRC cells with PMA-activated U937 cells (Forssell et al., 2007). In contrast, the proliferation of CT26 colon carcinoma cells was stimulated when the cells were co-cultured with macrophages derived from the peritoneal cavity (Luput et al., 2017). These conflicting observations may stem from the different origins of macrophages and suggest that the *in vitro* experiments do not reproduce the exact features of TAMs in CRC. A previous study revealed that TAMs differentiated from monocytes induced by CRC tumor cells had enhanced the expression of chemokines, antigen presentation, and T-cell co-stimulation molecules, thereby promoting T-cell antitumor responses (Ong et al., 2012). In 2020, a systematic review and meta-analysis summarizing 27 studies with 6,115 patients indicated that the role of TAMs depended on the infiltration location and mismatch repair (MMR) condition (Li et al., 2020). In that study, better 5-year overall survival (OS) was related to a high density of TAMs in the tumor, which was more prominent in MMR-proficient patients (Li et al., 2020). Stratification by TAM infiltration location revealed that a high density of CD68 + TAMs in tumor stroma and tumor islet plus stroma (but not in tumor islet cells) predicted a favorable OS (Li et al., 2020). Other studies supported these results. For example, Forssell and colleagues found a high density of CD68 + macrophages along the tumor front to be a good prognostic marker for colon cancer (Forssell et al., 2007). Waniczek et al. (2017) suggested that intense M2 macrophages (CD68 + iNOS-) infiltration within the tumor stroma was associated with shorter disease-free survival (DFS) and OS. In contrast, intense infiltration at the tumor edge and the surrounding tissues was associated with a lower recurrence rate (Waniczek et al., 2017). In resected colorectal liver metastases stained for CD68, a high density of TAMs correlated with longer DFS (Cavnar et al., 2017).

Macrophages are known to polarize, heading to either the M1 or M2 phenotypes. M1 is a proinflammatory phenotype, whereas M2 is anti-inflammatory. In cancer, several lines of research have suggested that M2 promotes tumor progression, metastasis, and angiogenesis (Ruffell et al., 2012). In most cases, TAMs are thought to be M2 and related to poor prognosis (Hao et al., 2012); however, the scenario is more complex in colon cancer. In addition, M2 also promotes metastasis in various ways, including MMP-9 expression (Vinnakota et al., 2017), miRNA-containing exosome secretion (Lan et al., 2019), and regulation of epithelial–mesenchymal transition (Lee et al., 2020). Immunohistochemistry (IHC) of CRC samples verified these results. Patients with a high CD206/CD68 ratio had significantly poorer DFS and OS (Feng et al., 2019a). Among 232 patients with CRC, Kaplan–Meier analysis revealed that M2/M1 >3 was related to significantly worse DFS and OS (Lee et al., 2020). Nonetheless, the dominant phenotype of TAMs in colon cancer is debatable, and the balance of M1/M2 changes in different periods in sporadic CRC resulting from the adenoma–carcinoma sequence (Isidro and Appleyard, 2016). Edin and colleagues found that when exposed to RKO, SW480, and Caco2 (i.e., CRC cell line–derived conditioned medium), the TAMs resembled the morphology and cell surface phenotype of M2 macrophage populations, but they showed a “mixed” M1/M2 macrophage phenotype with respect to cytokine and chemokine expression patterns (Edin et al., 2013). Following the former results, a study in 2018 reported the differentiation of monocytes into a mixture population of M1/M2 induced by colon cancer–derived conditioned medium (Sawa-Wejksza et al., 2018). Another study analyzed genes expressed by the TAMs and mapped them into biological functions to see whether TAMs were pro- or anti-inflammatory. Those genes were involved in inflammation (18%), differentiation (18%), chemotaxis (8%), MHC class II antigen presentation (3%), and phagocytosis and endocytosis (2%) (Ong et al., 2012), thus revealing a proinflammatory phenotype. These results suggested that TAMs in colon cancer cannot be simply identified as M1 or M2 phenotype. It is still useful, however, to consider that in various functional states, where M1 and M2 are extremes, re-educating TAMs to the M1 phenotype may be an efficient anticancer strategy.

Wnt signaling is essential in the proliferation and innate immune function of macrophages. Combretastatin A-1 phosphate downregulates the Wnt/β-catenin pathway and induces macrophages (Mao et al., 2016). Wnt3a promotes proliferation of macrophages, while blockade of β-catenin signaling reverses the effect (Feng et al., 2018a). The Wnt5a-NF-κB (p65) pathway is normally kept in a homeostatic state to support survival and the innate immune response of macrophages (Naskar et al., 2014). In addition, Wnt5a signaling enhances fz5-dependent internalization of nonpathogenic (Maiti et al., 2012) and pathogenic bacteria (Jati et al., 2018) and killing of the latter (Jati et al., 2018), whereas Wnt7a inhibits phagocytosis (Wallace et al., 2018).

The Wnt pathway is reported to regulate the pro- or anti-inflammatory phenotype alteration of macrophages. Wnt3a (Feng et al., 2018b; Yuan et al., 2020) or Wnt5a (Feng et al., 2018a) treatment could alter cytokine-stimulated macrophage polarization, thereby predisposing macrophages to the M2 phenotype. LPS-induced proinflammatory cytokine expression in macrophages was abolished after β-catenin knockdown (Fang et al., 2018). Wnt5a signaling, to which Fzd5 and camkii contribute, stimulated the release of proinflammatory cytokines in macrophages (Pereira et al., 2008). Activation of the Wnt5a/JNK1 pathway significantly increased the expression of TNF-α and IL-6 in pulmonary macrophages (Zhu et al., 2018). Wnt7a inhibited classical markers on macrophages in differentiation and polarization, and affected cytokine secretion from MDM (Wallace et al., 2018).

Wnt signaling in TAMs is complex. Macrophages express Wnt protein and frizzled receptors simultaneously (Blumenthal et al., 2006), which means that the Wnt protein is derived from both tumor cells and TAM. Both could trigger the Wnt pathway in TAMs, indicating the existence of a complicated regulation network. The role of Wnt signaling in TAMs has been partly revealed. Wnt signaling was significantly activated in M2-like TAMs (Yang et al., 2018; Sarode et al., 2020), and activation of Wnt/β-catenin signaling drove M2 polarization in TAMs (Raghavan et al., 2019). These results also were verified in human samples. High expression of Wnt5a in the tumor was significantly associated with intense anti-inflammatory CD163 + TAMs, but not with CD68 + TAMs (Bergenfelz et al., 2012). Accumulation of nucleus-located β-catenin was positively correlated with M2-like TAMs in human hepatocellular carcinoma (HCC) samples (Yang et al., 2018). Wnt3A expression was higher in HCC samples strongly stained for CD163 than in CD163-weakly stained samples (Tian et al., 2020). There are still unrevealed mechanisms, however, and it is still unknown how Wnt signaling in TAMs can be modified to benefit antitumor therapy.

The role of BCL9-driven Wnt signaling in macrophage polarization and how suppression of BCL9 affects tumor immune microenvironment remain unclear. In this study, we used single-cell sequencing technology to reveal the changes in TAMs in TME in genetic depletion and pharmacological inhibition of *Bcl9*.

## Materials and Methods

### Chemicals, shRibo Nucliec Acids and Cells

hsBCL9_CT_-24 was produced by AnaSpec, CA, according to previous protocols (Feng et al., 2019b). The synthesis and purification of peptides were evaluated using analytical high-performance liquid chromatography (HPLC) and mass spectrometry (MS). hsBCL9_CT_-24 was dissolved into 10 mmol/L; both were diluted before assay. pGIPZ (inducible with doxycycline)-based lentiviral shRNAs for mouse Bcl9 shRNA#5 (V3LMM_429161) and non-targeting shRNA were obtained from Open Biosystems/GE Dharmacon. The non-targeting (NT) lentiviral shRNA was constructed to be a negative control expressing no substantial homology to any mammalian transcript. Cell line CT26 cells (ATCC) were cultured according to the supplier’s recommendations. All cell lines used are listed using the official cell line name and its Research Resource Identifier (RRID) as available in the ExPASy Cellosaurus database [CT26 (RRID:CVCL_7254)]. All experiments were performed with mycoplasma-free cells.

### Tumor Specimens

At least four regions in each tumor were sampled. We obtained 12 tumor samples from 12 mice, respectively. Detailed information is presented in Supplementary Table S1. Male, BALB/c mice, 6–8 weeks old, were purchased from Shanghai Lingchang Biotechnology Co., LTD. Animals were housed under specific pathogen-free conditions (22 ± 1°C, 12/12 light/dark cycle) in Fudan University. All the procedures were performed according to protocols approved by the University’s animal care committee, along with the guidelines of “The Association for Assessment and Accreditation of Laboratory Animal Care International.”

### Tumor Size Measurement

Cultured CT26 colon cancer cells (ATCC) were harvested for subcutaneously (s.c.) inoculation (4 × 10^5^ cells in PBS) in the right flank region of BALB/c female mice (purchased from Charles River) at 6–8 weeks of age. Three randomized cohorts (*n* = 4) with tumor size between 30 and 50 mm^3^ were administered vehicle control, hsBCL9_CT_-24 (25 mg/kg, i. p., QD) or hsBCL9_CT_-24 (30 mg/kg, sc., QD) for 17 days after inoculation. In the CT26 combination experiment (*n* = 4) model, mice were grouped into four randomized cohorts and given hsBCL9_CT_-24 (25 mg/kg, i. p., QD), anti–PD-1 antibody (10 mg/kg, i. p., BIW), or a combination arm (hsBCL9_CT_-24 + anti–PD-1 antibody) *via* i. p. injection after tumor volume reached 30 mm^3^. Colo320-DM (ATCC) were harvested for subcutaneously (s.c.) inoculation (1 × 10^6^ cells in PBS) in the right flank region of BALB/c female nude mice (purchased from B&K Universal Group Limited) at 6–8 weeks of age. Two randomized cohorts (*n* = 4) with tumor size between 30 and 50 mm^3^ were administered vehicle control or hsBCL9_CT_-24 (15 mg/kg, i. p., QD) for 20 days after inoculation. Tumor volume was measured every other day using digital calipers and calculated on the basis of the following formula: tumor volume = (length × width^2^)/2. Murine body weights were recorded after tumor measurement. Tumor size of >1000 mm^3^ was set as the endpoint, and tumor/organ samples were collected from selected mice at the end of the study.

### Specimen Processing

Fresh tumors from mice were surgically resected and collected into MACS Tissues storage solution (130-100-008, Miltenyi Biotec). The samples were immediately transferred to the Fudan laboratory. For further processing, tissues were minced into <1 mm^3^ on ice, shifted to a C tube (130-093-237, Miltenyi Biotec), and enzymatically digested by MACS Tumor Dissociation Kit (130-095-929, Mitenyi Biotec). The obtained suspension was filtered through a 40 μm cell strainer (Falcon) and washed by RPMI 1640 (C11875500BT, Gibco). Subsequently, erythrocytes were removed using 2 ml the Red Cell Lysis Buffer (555899, BD biosciences) and live cells were enriched by a Dead Cell Removal Kit (130-090-101, Miltenyi Biotec). After re-suspension in RPMI 1640 (C11875500BT, Gibco), a single-cell suspension was obtained. Cell viability, which needs to be >90% for library construction, was checked by Trypan blue (15250061, Gibco) staining.

Samples were sequenced using the BGISEQ-500 platform at The Beijing Genomics Institute (BGI) for Genomics and Bioinformatics. Raw counts were then normalized to fragments per kilobase of transcript per million mapped reads (FPKM). Cells with genes between 500 and 50,000, UMI numbers less than 30,000, and mitochondrial content less than 20% were selected. Cells identified as doublets or multiplets based on gene expression signatures, which had more than one highly expressed cell population-specific markers, were filtered out. Filtered data were normalized using a scaling factor of 10,000, and data was log transformed. The sequencing coverage and quality statistics for each sample are summarized in Supplementary Table S2.

### 10 × Library Preparation and Sequencing

A single-cell suspension was prepared and ran on a Chromium Single-Cell Platform (see *Materials and Methods*). Before running on a Chromium Single-Cell Platform (10 × Genomics Chromium™), the cell concentration was adjusted to 700–1200 cells/ul. A 10 × library was generated following the manufacturer’s protocol of 10 × genomics Single-Cell 5′ Gel Bead Kit. The clustering was conducted on a cBot Cluster Generation System with TruSeq PE Cluster Kit v3 implemented the clustering. Qubit was used for library quantification. The final library was sequenced on an Illumina HiSeq3000 instrument using 150-base-pair paired-end reads.

### Reduce Dimensionality Analysis

The number of unique molecular identifier (UMI) sequences of high-quality per cell was counted and normalized to the median of the total UMI of all cells using the median normalization process. Means of the Principal component analysis (PCA) reduction dimension were used to evaluate the similarity between cells. The expression trend of cellular genes is positively correlated with the sample distance. The largest variance explained results from using t-distributed stochastic neighbor embedding (t-SNE) or Uniform Manifold Approximation and Projection (UMAP) to visualize the single-cell clustering for the PCA’s top 10 principal components. In the t-SNE presentation method, the sample distance is recounted to neighbor’s conditional probability neighbor fitting according to the Student T-distribution in the high-dimensional space. The sample can display a divided cluster in the low dimensional space.

### Pathway and Functional Annotation Analysis

Pathway annotation and enrichment was performed through Gene Set Enrichment Analysis (GSEA) (Subramanian et al., 2005). MsigDB is a database resource for investigating the high-level functions and effects of the biological system (Liberzon et al., 2011). Pathways with a Q value ≤0.05 were considered to be significantly enriched. Based on The Gene Ontology database, we conducted functional annotation, including biological process, cellular component, and molecular function classifications.

### Tumor-Associated Macrophages-To-Natural Killer (NK) Score

Use Eq. 1 to calculate the TAM-to-NK Score of each cell or each patient.

$$\text{TAM}-\text{to}-\text{NK}\;\text{Score}=\frac{\text{GSVA}\left(\text{TAM}\_\text{type}\_\text{list}\right)}{\text{GSVA}\left(\text{NK}\_\text{type}\_\text{list}\right)}$$ (1)

In the Eq. 1, GSVA () indicated Gene Set Variation Analysis; TAM type gene list (TAM_type_list): *APOE, C1QA, C1QB, C1QC, CCL12, CCL6, CCL8, LYZ2, PF4, WFDC17, CSF1R,* and *CXCL14*; NK-like non-functioning type gene list (NK_type_list):. *AW112010, CCL5, CD3E, CD52, GZMA, GZMB, GZMC, IL2RB, LTB,* and *NKG7.*

### Gene Set Variation Analysis and Metascape Analysis

Based on cell population expression data, we obtained the average expression of each gene of the corresponding cell in each sample. GSVA analysis was conducted based on the resulting expression data of the above two types of cells. After performing GSVA Z-score analysis by the gene list above, we obtained the GSVA Z-score of each pathway in each sample. The GSVA score data of the above four types of cells were calculated in the limit package in R. Metascape analysis is carried out according to the published method (Zhou et al., 2019).

### Gene Prognostic Performance in The Cancer Genome Atlas Sample

Standardized TCGA datasets are derived from Xena Functional Genomics Explorer (https://xenabrowser.net/). Maxstat (maximally selected statistical status) algorithm is used to distinguish the high and low TAMtoNK Score. Kaplan-Meier curve is used to analyze the overall and disease-free survival of the patient population. Log-Rank *p* value and HR were also established using the same software as survival analysis.

### Statistical analysis

Statistical significance was determined using 2 way ANOVA. (GraphPad Prism 8). The *p*-values were then calculated using Tukey’s multiple comparisons test. *p* < 0.05 was considered statistically significant.

## Results

### The Effect of bcl9 Inhibitors on Tumor Growth

**FIGURE 1:**
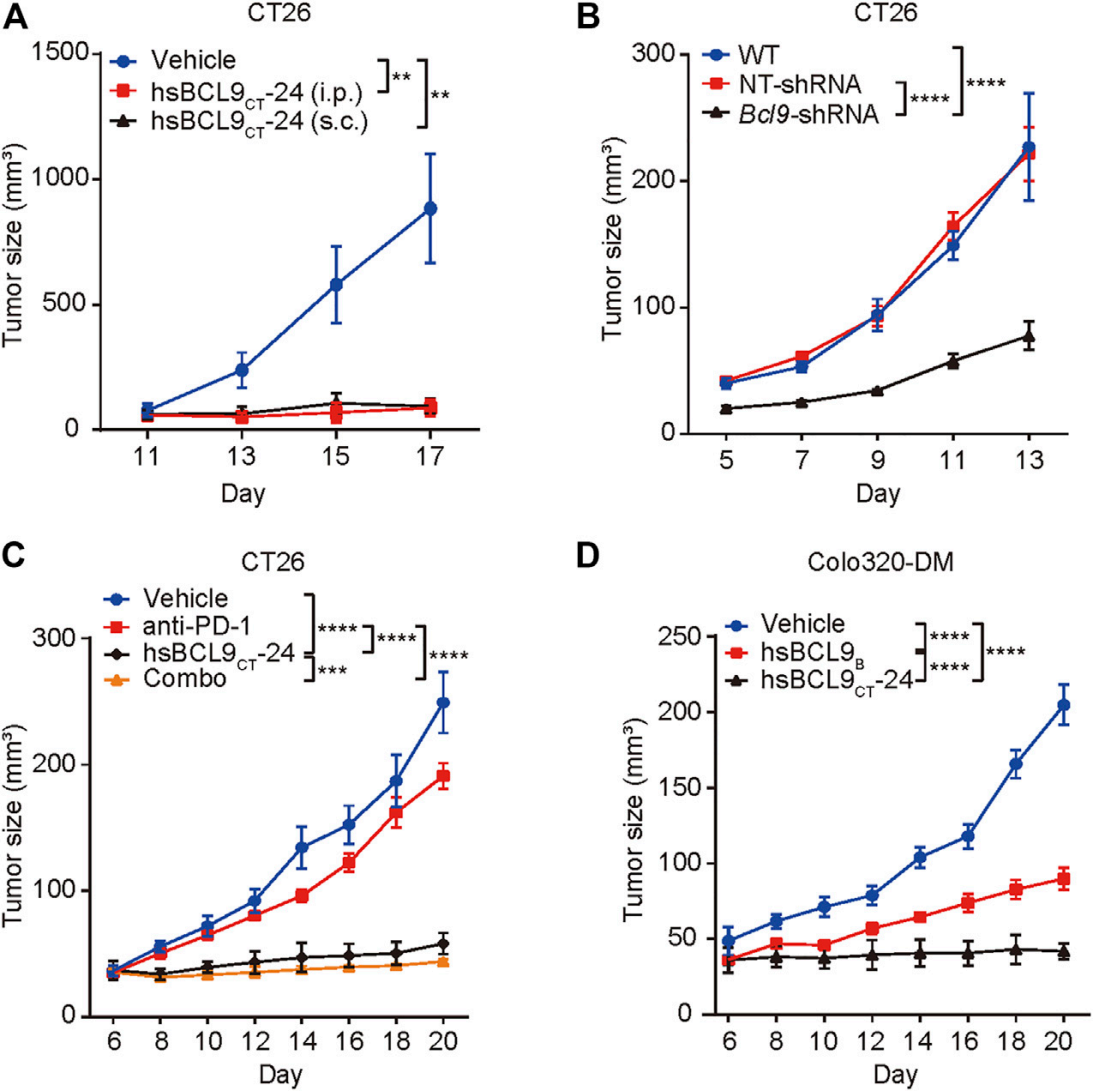
The effect of bcl9 inhibitors on tumor growth.**(A)** BALB/c mice were inoculated with CT26 cells *via* single flank implantation and treated with hsBCL9_CT_-24 (ip., 25 mg/kg, QD) or hsBCL9_CT_-24 (sc.,30 mg/kg, QD) as indicated after tumor volume reached 30 mm^3^ (*n* = 4 per cohort). **(B)** CT26 cells which were transduced with NT-shRNA or *Bcl9*-shRNA were inoculated in BALB/c mice (*n* = 5 per cohort). **(C)** Combination treatment of hsBCL9_CT_-24 and anti–PD-1 Ab resulted in almost complete regression in the CT26 model. BALB/c mice were inoculated with CT26 cells *via* single flank implantation and treated with hsBCL9_CT_-24 (ip., 25 mg/kg, QD), anti–PD-1 Ab [ip.,10 mg/kg, twice weekly (BIW)], and hsBCL9_CT_-24 + anti–PD-1 Ab as indicated after tumor volume reached 30 mm^3^ (*n* = 4 per cohort). **(D)** Colo320-DM cells were inoculated in BALB/c nude mice before treatment with vehicle control or hsBCL9_CT_-24 (ip.,15 mg/kg, QD) as indicated after tumor volume reached 30 mm^3^ (*n* = 4 per cohort). Significance were tested by 2-way ANOVA for experiments performed in triplicate, and each experiment was repeated three times, **p ≤ 0.01, ***p ≤ 0.001, ****p ≤ 0.0001.

The CT26 tumor cells were implanted s. c. into the right flanks of BALB/c mice; tumor-bearing animals were treated with vehicle control or hsBCL9_CT_-24 (ip., at 25 mg/kg or sc.,30 mg/kg) for 17 days hsBCL9_CT_-24 significantly reduced tumor volume relative to control throughout the study period (Figure 1A) and genetic knockdown of Bcl9 in CT26 also significantly reduced tumor growth in compared with non-targeting RNA (NT -shRNA) (Figure 1B). CT26 has low response to anti-PD-1 Ab treatment. We therefore examined whether treatment using hsBCL9_CT_-24 could improve this response through immune-editing functions. In CT26 mouse model, combinatory treatment with hsBCL9_CT_-24 and anti–PD-1 Ab markedly reduced tumor growth (Figure 1C) And hsBCL9_CT_-24 was assessed in the mouse CRC model established in both immunocompetent (regular BALB/c) and immunodeficient (nude) mice. In nude mice, hsBCL9_CT_-24 exhibited a comparable potency against the growth of Colo320-DM (Figure 1D).

### Single-Cell RNA-Seq of Mouse CT26 Tumor With Pharmacological Inhibition of Bcl9 and Genetic Depletion of Bcl9

**FIGURE 2:**
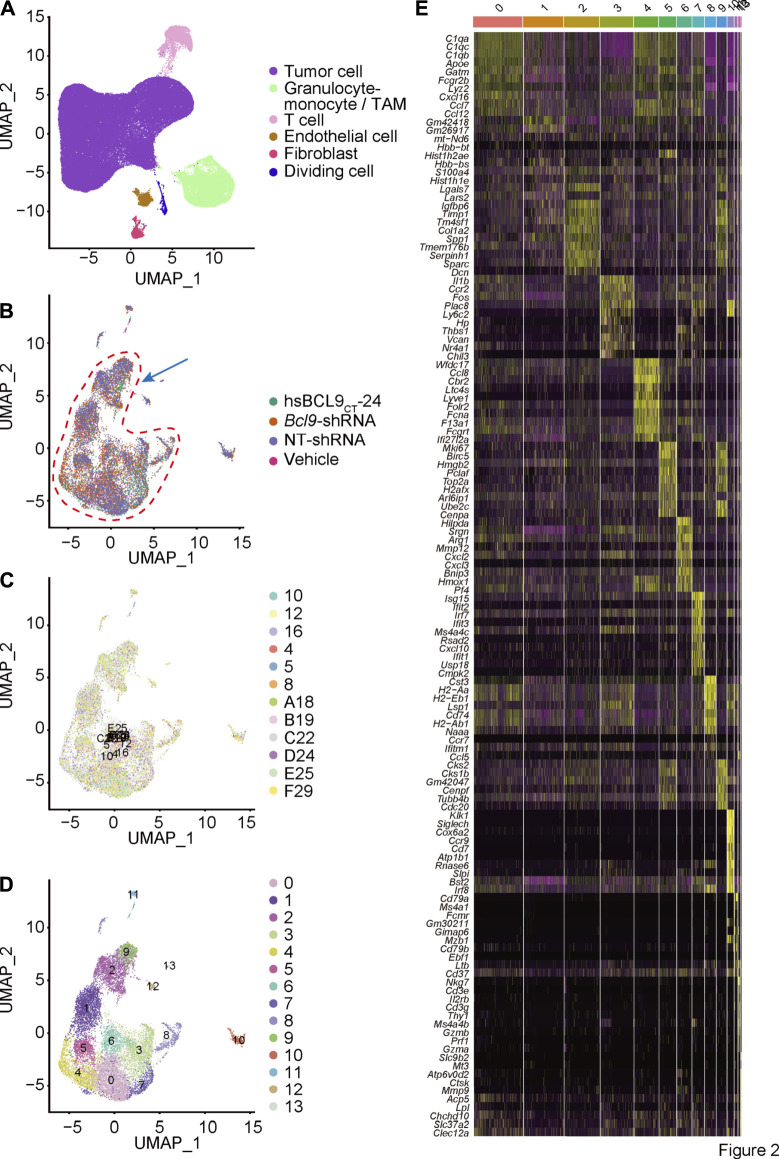
Single cell cluster analysis of BCL9 perturbed CT26 murine tumor. **(A)** UMAP mapping and cell type. Re-cluster analysis of Granulocyte-monocyte (Step 1 re-clustering). **(B)** UMAP mapping and groups (vehicle, hsBCL9_CT_-24, NT-shRNA, *Bcl9*-shRNA), the arrow shows the cell populations with difference. **(C)** UMAP mapping and clusters. **(D)** UMAP mapping and three clusters (clusters 2, 9 and 12) with differences (Used for Step 2 re-clustering). **(E)** Heatmap of the 13 clusters from Step 1 re-clustering. Columns, individual cells; rows, genes (Top10).

To investigate the heterogeneity of murine CT26 tumor treated with hsBCL9_CT_-24 and genetically depleted *Bcl9*, we conducted single-cell mRNA sequencing in 12 samples (see *Materials and Methods*). After integrating the data from four different groups of treatment, including hsBCL9_CT_-24 (hsbcl9), vehicle (veh), *Bcl9*-shRNA (KD), Non-targeting shRNA (NT), we used an algorithm built in the Seurat package to remove batch effects. After completing the principal component analysis of genes, UMAP, which is a dimensionality reduction method based on manifold analysis, grouped the cells together according to the similarity of gene expression (Figure 2A). Through the clustering analysis of all cells, we found that these cells can be divided into eight clusters corresponding to tumor cells, tumor-associated monocytes, TAMs, tumor-associated endothelial cells, fibroblasts, and T cells (Figure 2A), these classifications are based on known markers or bioinformatics research (Wei et al., 2020). To study the potential differences in TAMs, we extracted the TAM population based on the cell information we previously reported (Wei et al., 2020), and performed re-clustering analysis (Step 1 re-clustering; Figures 2B–E). Most cells showed a notable overlap in the reduced dimension maps between different samples or treatment groups (Figures 2A–C) large number of cells continuously distributed topological structure (cells circled by red dashed line; Figure 2B). According to the marker gene of these clusters, they could be classified as the main population of TAMs (Figures 2D,E) (Shalhoub et al., 2011). The next section includes an analysis of the main population of TAMs.

### Macrophage Polarization: Cellular Landscape Difference Between M0, M1, and M2

**FIGURE 3:**
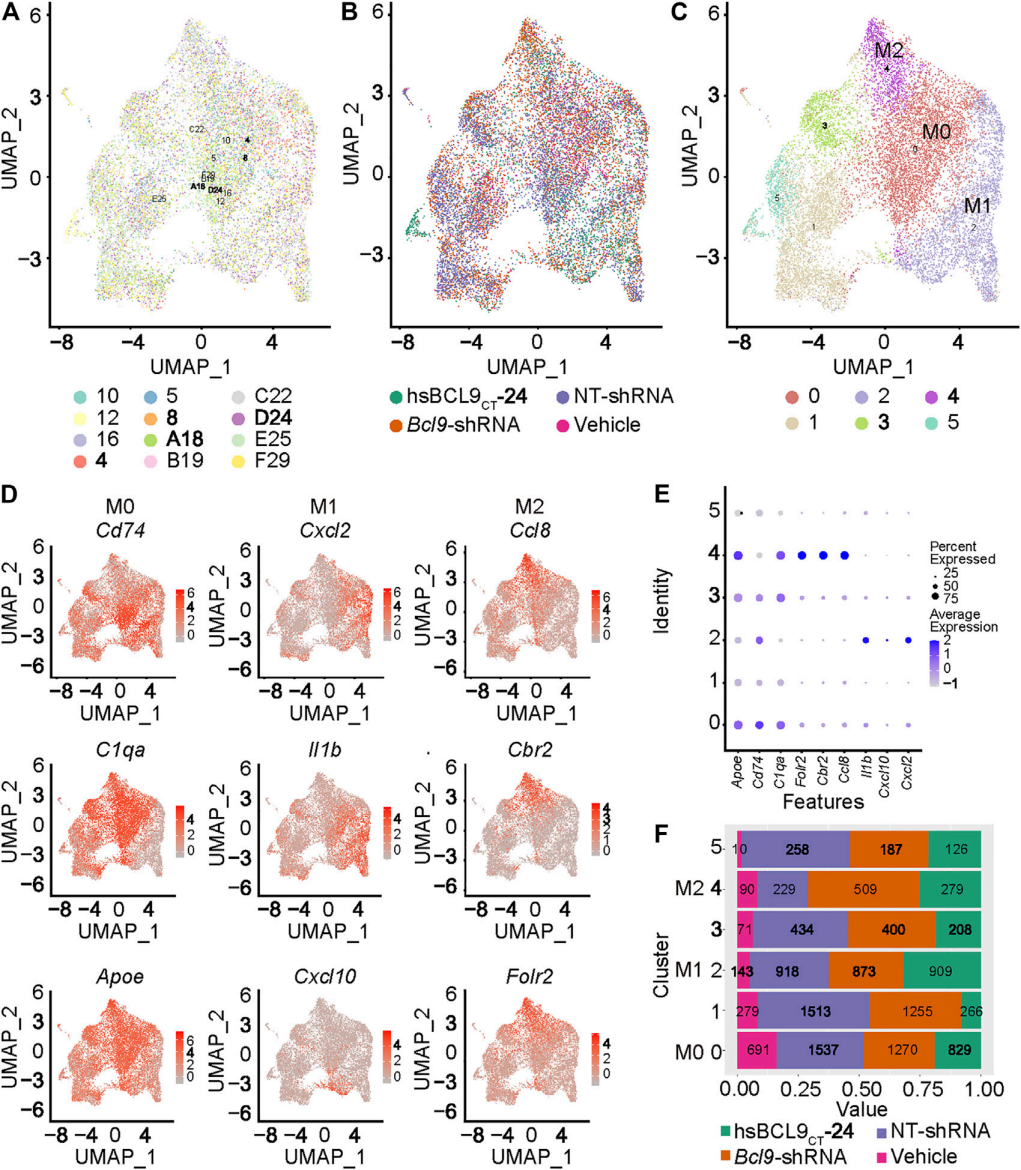
Population of M0, M1 and M2 in the TAM of mouse CT26 tumor. Re-cluster (Step 2 re-clustering) analysis of TAM (The population circled in red dash in Figure 2). **(A)** UMAP mapping and clusters and samples. **(B)** UMAP mapping and groups (vehicle, hsBCL9_CT_-24, NT-shRNA, *Bcl9*-shRNA). **(C)** UMAP mapping and clusters. **(D)** UMAP mapping and M0, M1 and M2 marker Gene expression level. **(E)** Bubble chart of TAM clusters with M0, M1 and M2 marker Gene expression level. **(F)** Barchart of cell proportions of different groups in TAM clusters, the number is the actual number of cells.

To further study the classification and biological characteristics of the main population of TAMs, we re-clustered the cells circled in red in Figure 2B. The results (Figures 3A,B) showed that the cells from different samples or treatment groups were all merged together, reflecting the successful removal of batch effects. Furthermore, we used the k-nearest neighbors algorithm to cluster cells based on the difference in gene expression (Figure 3C). The main population of TAMs could be classified into six populations, among which the three main populations (0, 2, and 4) were topologically connected and occupied the vast majority of the TAM group (Figure 3C). This geometric topological connection implies a potential cell population relationship. Furthermore, the analysis of marker genes of these populations proved that clusters 0, 2, and 4 expressed high amounts of marker genes at different stages of macrophage polarization. For example, cluster 0 expressed the known markers of M0 (*Cd74*, *C1qa*, and *Apoe*), and cluster 2 expressed M1 markers (*Cxcl2*, *Il1b*, and *Cxcl10*), whereas cluster 4 expressed M2 markers (*Ccl8*, *Cbr2*, and *Folr2x*) (Figures 3D,E). The differential expression of these genes proved that clusters 0, 2, and 4 represented the three states of macrophage polarization.

**FIGURE 4:**
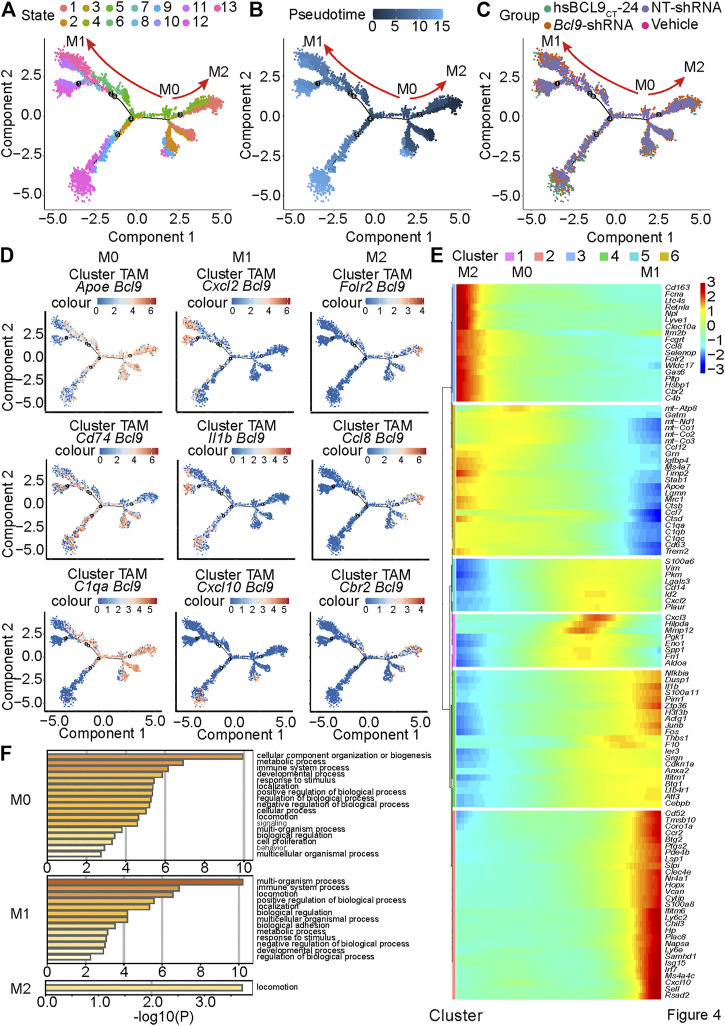
Pseudotime analysis reveals the trajectory of M0, M1 and M2 in mouse CT26 tumor. Pseudotime analysis on the TAM cells of clusters 0, 2 and 4 in Figure 3C and **(A)** States; **(B)** Pseudotime; **(C)** groups (vehicle, hsBCL9_CT_-24, NT-shRNA, *Bcl9*-shRNA); **(D)** M0, M1 and M2 marker Gene expression level; **(E)** trend of the gene expression generated by the BEAM function along pseudotime. **(F)** Metascape biological processes enrichment analysis by using gene clusters abtained from BEAM function.

To further prove the existence and differentiation process of the three states of macrophage polarization (M0, M1, and M2 polarization) in CT26 tumor, we extracted clusters 0, 2, and 4 (from Figure 3C) to analyze the trajectory using the DDR-Tree algorithm included in the Monocle package. As shown in Figures 4A–C, the cell population was projected on a dendritic structure. State analysis, pseudotime analysis, and classification based on treatment groups are displayed in Figures 4A–C. Further analysis showed that classic marker genes of M0, M1, and M2 were obviously polarized on the trajectory (Figure 4D). The M0 markers (*Cd74*, *C1qa*, and *Apoe*) tended to be expressed in the middle of the trajectory, and the M1 markers (*Cxcl2*, *Il1b*, and *Cxcl10*) tended to be expressed in the direction of the right arrow. The M2 markers (*Ccl8*, *Cbr2*, and *Folr2x*) tended to be expressed in the direction of the left arrow (Figure 4D). This was consistent with the classic M0, M1, and M2 marker conversion paradigm (Shalhoub et al., 2011; Yuan et al., 2015; Macciò et al., 2020).

To study the overall gene expression trend of the cell population along pseudotime, we used the BEAM function to analyze the differential gene expression along time (Figure 4E). The results showed that some different genes were enriched in different stages of the differentiation process (Figure 4E). The genes enriched in the middle and right ends may participate in the biological processes related to M0 and M1, and it is expected that the genes enriched in the left end participate in the biological processes related to M1 and M2. We analyzed these gene sets by Metascape to better understand the associated biological processes (Zhou et al., 2019) (Figure 4F). The results showed that gene sets that favored M0, M1, or M2 participated in immune and inflammation-related signal pathways to varying degrees. These conclusions can provide inspiration for further mechanism research.

### Differential Gene Expression and Signal Pathway Enrichment Analysis of Mouse CT26 Tumor With Bcl9 Deprivation in the Tumor-Associated Macrophages Subgroups

**FIGURE 5:**
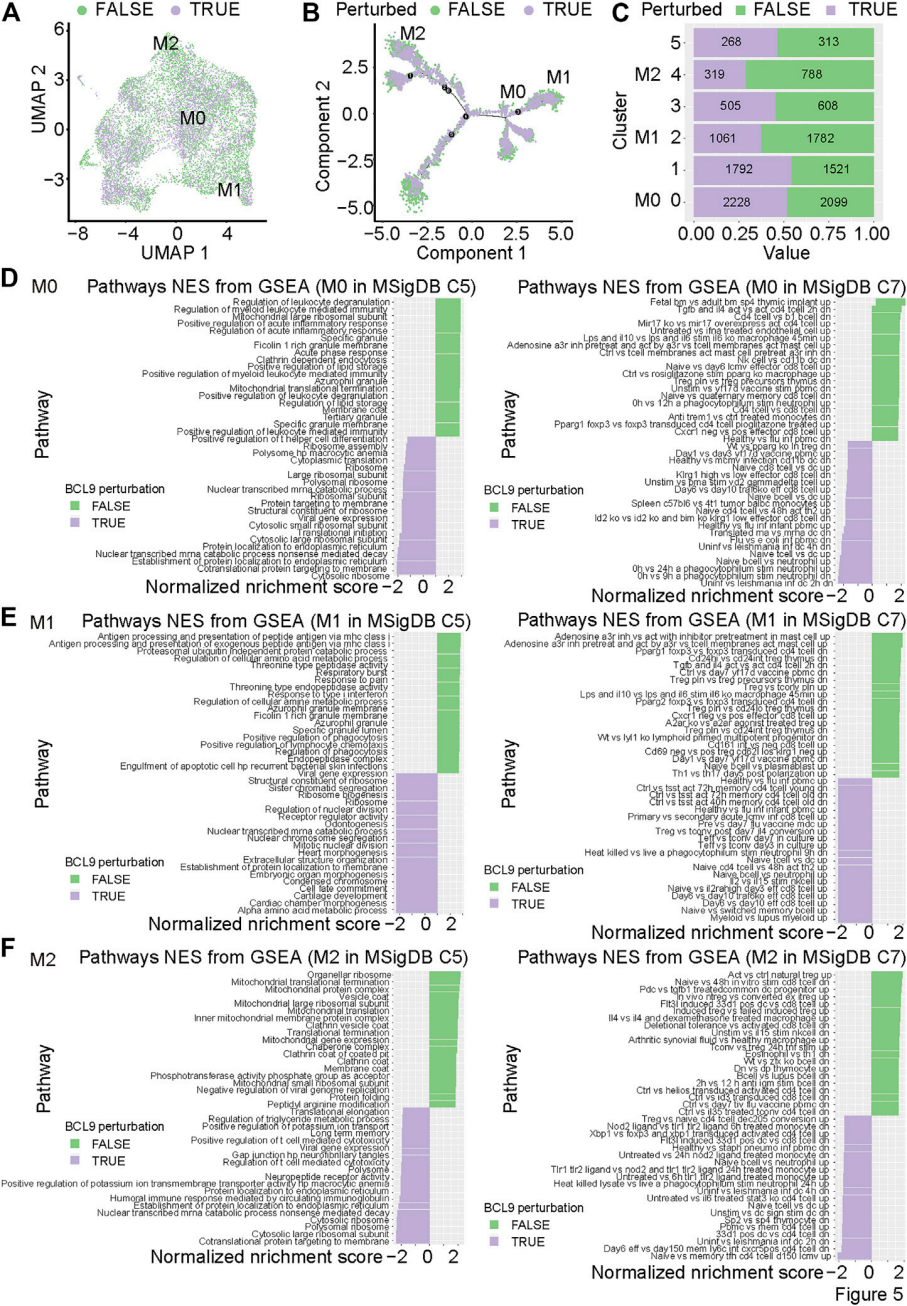
The perturbation of *Bcl9* inhibits the differentiation and function of M0, M1 and M2 in CT26 tumor. **(A)** UMAP mapping of TAM (Figure 3A) and perturbation of *Bcl9* or not; **(B)** trajectory of M0, M1 and M2 (Figure 4A) and perturbation of *Bcl9* or not; **(C)** Barchart of cell proportions from perturbation of *Bcl9* in TAM clusters, the number is the actual number of cells. GSEA analysis for M0 **(D)**; M1 **(E)**, and M2 **(F)** TAM cells, MsigDB C5 (All gene sets derived from Gene Ontology) is used on the left, and MsigDB C7 (immunologic signature gene sets) is used on the right.

To further study the effect of *Bcl9* deprivation of M0, M1, and M2 TAMs, we classified the group (hsbcl9, KD) with *Bcl9* deprivation into *Bcl9* perturbed True group, and their controls (veh, NT) into Bc9 perturbed False group. UMAP clustering and trajectory analysis showed no obvious difference between the two groups (Figures 5A,B). Comparison of the number of cells in different clusters, however, revealed that the proportion of *Bcl9* perterbed True cells in M2 was significantly lower than that of *Bcl9* perterbed False cells. The decrease in KD in the shRNA group was the most obvious, and the proportions were roughly the same in M0 (Figure 5C and Figure 3F). This result showed that the deprivation of *Bcl9* may have interfered with the differentiation of M0 to M2. M2 cells are widely regarded as “bad” TAMs, which promote tumor growth (Yuan et al., 2015; Macciò et al., 2020). Nevertheless, there were still many M1 and M2 cells in the *Bcl9* perterbed True group.

To further study the genes that are differentially expressed in M0, M1 and M2, and understand which biological processes are affected by the deprivation of *Bcl9*, we performed Gene Set Enrichment Analysis (GSEA) analysis on TAMs. As shown in Figures 5D–F, we performed GSEA analysis on *Bcl9*-deprived and non-deprived cell populations from M0, M1, or M2, using the overall biological process database MsigDB C5 and immune-related signaling pathway database MsigDB C7 (Subramanian et al., 2005; Liberzon et al., 2011). The results showed that the signaling pathways related to immune and inflammatory response in M0 and M1 were significantly enriched in the *Bcl9* perterbed False group, but there was no obvious enrichment in M2. This indicated that *Bcl9* deprivation may have had a strong inhibitory effect on the inflammatory response caused by M0 and M1 cells. Inflammation is one of the most important factors that causes tumor development and prognosis (Coussens and Werb, 2002).

To further clarify the upstream transcription factor regulation mechanism related to these biological processes, we used MsigDB C3 to perform transcription factor analysis. The results are shown in Supplementary Figure S1. In the *Bcl9* perterbed True group, Period Circadian Regulator 1 (Per1) target genes were highly enriched in M0, M2, and M2, and the function of Per1 was in circadian regulation (Ono et al., 2017). This result showed that the deprivation of *Bcl9* may disrupt the circadian rhythms of M0, M2, and M2.

### Depletion of Bcl9 Inhibits the Delineation From Tumor-Associated Monocytes to NK Cells

To further study the changes of TAMs to discover new cell populations, we again checked the cell classification from Figure 2B. Although most cells from different treatment groups (hsbcl9, veh, KD, NT) were merged together, the clusters 2, 9, and 12 shown in Figure 2B were not merged well. Topologically, some cells from cluster 12 were strongly related to cells from cluster 2 and were located at the junction of these two clusters (Figure 2B, indicated by blue arrow). We showed that these cells were the most responsive and sensitive to *Bcl9* deprivation. According to the difference in genes from Figure 2E, we confirmed that most clusters shown in Figure 2D highly expressed the markers of monocytes (*C1qb*, *C1qc*, and *C1qa*), which are the precursor cells of TAMs (Tran et al., 2017), whereas cluster 12 highly expressed the markers of natural killer cells and NK cells (*Nkg7*, *Il2rb*, and *Cd3e*). Coupled with the topological correlation of these cell populations, we inferred that cluster 12 belonged to the NK cells delineated from monocytes (Chen et al., 2015).

**FIGURE 6:**
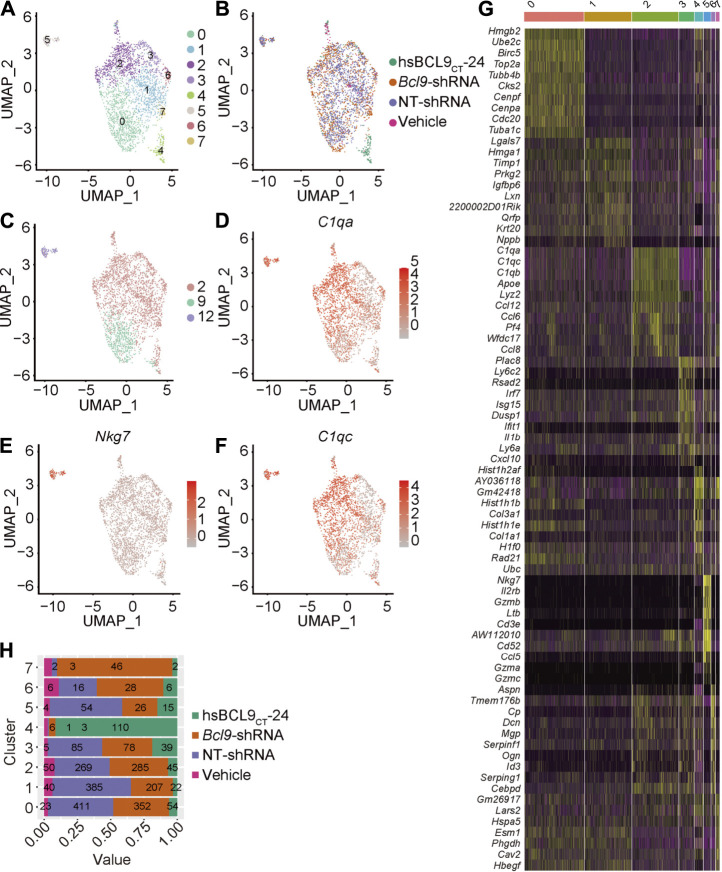
Re-cluster (Step 2 re-clustering) analysis of TAM (monocytes) and delineated NK-like non-functioning cell. **(A)** UMAP mapping and clusters. **(B)** UMAP mapping and groups (vehicle, hsBCL9_CT_-24, NT-shRNA, *Bcl9*-shRNA). **(C)** UMAP mapping and Step 1 re-clustering clusters labeling. **(D–F)** UMAP mapping and monocytes and NK cell marker gene expression level (C1qa, C1qc and Nkg7). **(G)** Barchart of cell proportions of different groups in Step 2 re-clustering, the number is the actual number of cells. **(H)** Heatmap of the 7 clusters from Step 2 re-clustering. Columns, individual cells; rows, genes (Top10).

To further verify that cluster 12 was indeed a delineated NK cell population, we performed the secondary re-clustering analysis to clarify the whole process of cell delineation. We extracted the TAM cells from Step 1 re-clustering (clusters 2, 9, and 12) shown in Figure 2D, and performed the second step of re-clustering (Step 2 re-clustering). As shown in Figures 6A–C, Step 2 re-clustering divided the cells into several clusters. Clusters 4, 5, and 7 were topologically relevant, which indicated their potential delineation relationship. Further differential expression analysis showed that cluster 5 contained only the NK cell markers (*Nkg7*, *Il2rb*, and *Cd3e*), and cluster 2 contained two kinds of markers, both of monocytes and NK cells (*Nkg7*, *Il2rb*, *Cd3e*, *C1qb*, *C1qc*, and *C1qa*; Figures 6D–G), which could be regarded as a transition state of this delineation process. Further quantitative analysis (Figure 6H) proved that the populations of the cells of the deprived *Bcl9* group were enriched in clusters 4 and 7. This result showed that during the process of delineation from monocytes to NK-like non-functioning cells, it is very likely that cluster 4 and 7 are the blocking stages because of *Bcl9* deprivation.

### Depletion of Bcl9 Inhibits the Delineation From Tumor-Associated Monocytes to NK Cells, Which Depends on Transcriptional Regulation

**FIGURE 7:**
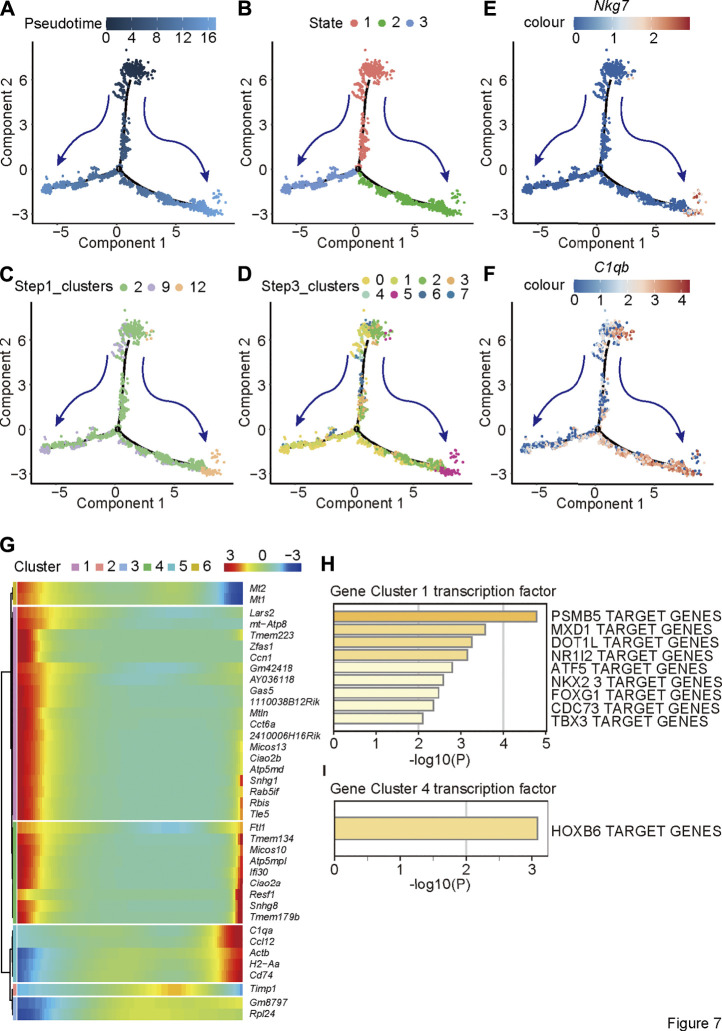
Pseudotime analysis reveals the trajectory of delineation from tumor-associated monocytes to NK-like non-functioning cells. Pseudotime analysis on the TAM cells of Step1 re-clustering (clusters 2, 9 and 12) and **(A)** Pseudotime; **(B)** States; **(C)** labeling by Step 1 re-clustering clusters **(D)** labeling by Step 2 re-clustering clusters; **(E,F)** monocytes and NK cell marker gene expression level (*C1qb* and *Nkg7*). **(G)** The trend of the gene expression generated by the BEAM function along pseudotime of Step1 re-clustering (clusters 2, 9 and 12) TAM population. **(H)** Metascape biological processes enrichment analysis by using gene clusters abtained from BEAM function.

To further study how *Bcl9* depletion inhibits the delineation from tumor-associated monocytes to NK-like non-functioning cells, we performed pseudotime analysis on the cells from Step 1 re-clustering (clusters 2, 9, and 12; Figure 6A). According to the results in Figures 7A–D, along pseudotime, cells delineated from state 1 to states 2 and 3.

Displaying clusters from Figure 6 on delineation trajectory, the un-delineated clusters (Step 2 re-clustering clusters 2 or Step 2 re-clustering clusters 4, 5, and 7) were in the more primitive stage of delineation (State 1)—that is, in the stage of monocytes—while cells at delineated clusters (Step 2 re-clustering clusters 12 or Step 2 re-clustering rest clusters) were at the stage (States 2 and 3) close to NK cells (Figures 7C,D). Nkg7 are C1qb were shown as two-stage markers at the two ends of the trajectory (Figures 7E,F). To further study the change in trend of gene expression in this process, we used the BEAM function to analyze the difference of the cell population along the pseudotime (Figure 7G). The results showed that the expression of NK cell markers (cluster 5) gradually increased, whereas the expression of other genes decreased (cluster 1) or first decreased and then increased (cluster 4). It is not clear to which signaling pathways two clusters of genes other than NK cell markers were related, but according to transcription factor analysis, the gene cluster with decreasing expression (cluster 1) was controlled mainly by the transcription factor PSMB5. The gene cluster that first decreased and then increased (cluster 4) was controlled mainly by the transcription factor PSMB5 and HOXB6.

### Low Tumor-Associated Macrophages-To-NK Score Predicts Poor Prognosis in Cancer Patients

**FIGURE 8:**
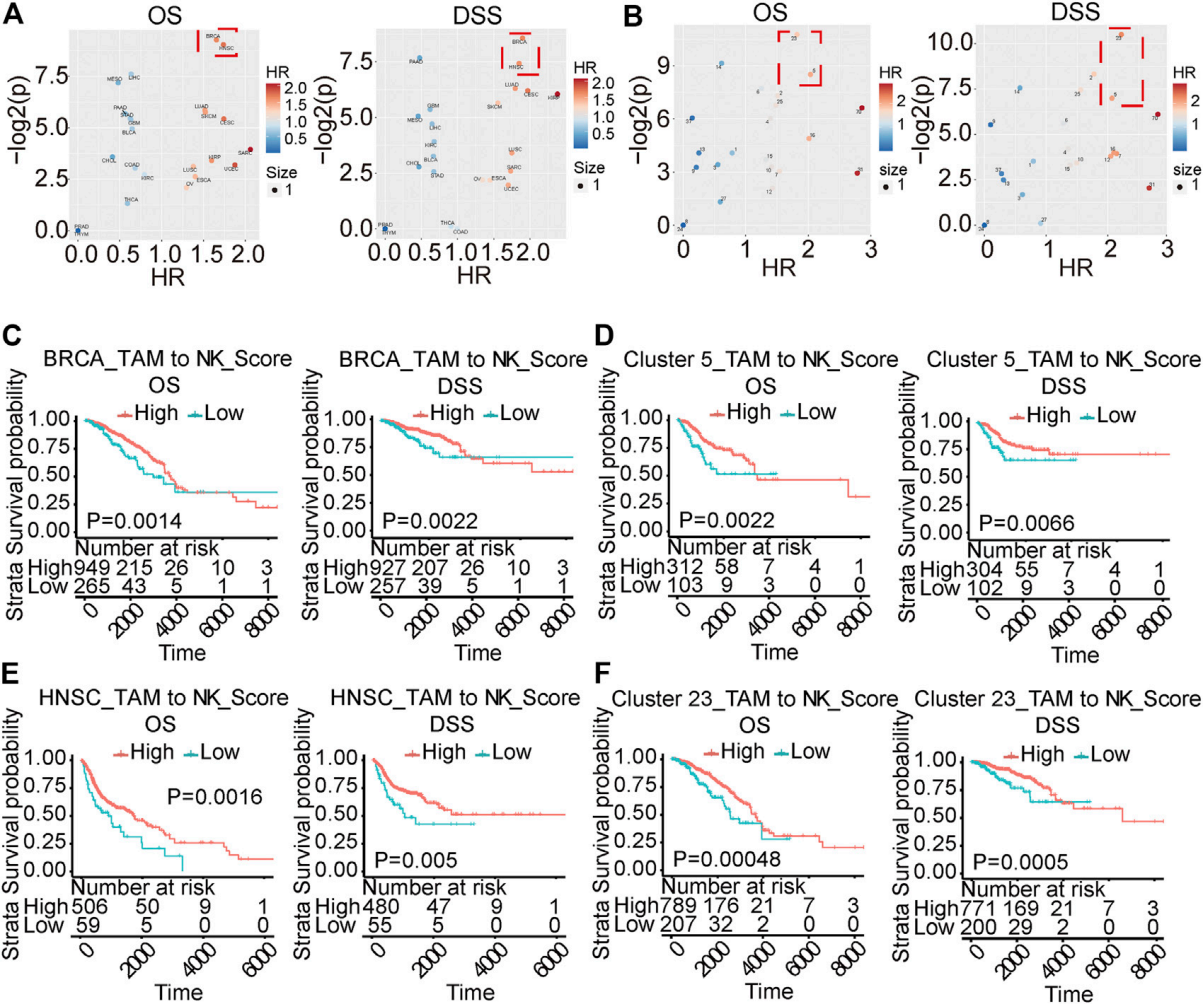
TAMtoNK Score predicts the prognosis of TCGA tumor patients. **(A,B)** Volcano chart of hazard ratio based on TAM-to-NK Score grouping of patients with different types of cancer or different clusters. **(C–F)** Survival analysis of TCGA tumor type or clusters with significant High hazard ratio. OS and DSS respectively represent overall survival and disease-specific survival rate.

Previous results suggested dynamic balance between the infiltrating monocytes and NK-like non-functioning cells in the tumor. To study the clinical significance of the transition between monocytes and NK-like non-functioning cells in tumors, we constructed a TAM-to-NK score to quantify the homeostasis between monocytes and NK-like non-functioning cells in tumors. The TAM-to-NK score quantified the degree of enrichment of the identified key marker genes in monocytes and NK-like non-functioning cells. To further verify that this score could predict the prognosis of cancer patients, we used it in the TCGA pan-cancer data set to estimate the TAM-to-NK scores of different patients. Next, we grouped the patients according to the TAM-to-NK scores, and conducted survival analysis to calculate the hazard ratio (HR). The results showed that low TAM-to-NK score predicted the poor prognosis of certain kinds of patients (Figures 8A,B). Among them, Breast invasive carcinoma (BRCA) and Head and Neck squamous cell carcinoma (HNSC) were the most prominent (Figures 8C,E). Because tumors classified according to their location of incidence have large gene expression pattern unpredictability, we previously reported a method to cluster the samples of the TCGA pan-cancer dataset according to their gene expression patterns (Wei et al., 2020). The advantage of this method is that it can eliminate the differences between the samples as much as possible, so that the comparison of specific gene sets or clinical prognosis are based on a highly consistent population. Then we used TAM-to-NK score to group patients in each cluster and conducted survival analysis. Low TAM-to-NK score predicted poor prognosis of patients from cluster 5 and cluster 23 with high significance (Figures 8D,F). Among them, cluster 23 was composed mainly of BRCA, and cluster 5 was composed mainly of BRCA, Uterine Corpus Endometrial Carcinoma (UCEC), and Cervical squamous cell carcinoma and endocervical adenocarcinoma (CESC). According to our previous reports, cluster 5 had marker genes *TYR* and *MLANA* (Wei et al., 2020). These results suggested that *TYR* and *MLANA* may be used as pathological criteria for prognostic prediction with TAM-to-NK score.

### Ligand-Receptor Interactions Analysis Between Tumor-Associated Macrophages and T Cells in the Tumor Microenvironment

To identify potential cell–cell interactions that are conserved across the two synergic tumor models, we scored potential ligand–receptor interactions across the cell types present within the TME. The interaction was determined by calculating the average expression of receptors and ligands. After calculating the score for each ligand and receptor, we averaged the interaction score of the tumor model to determine the conservative interaction. The macrophages interacted with T cells through the CCL3-CCR5, CAF1R-CSF1, and ICAM1-ITGAL to change the T-cell functions in hsBCL9_CT_-24 treated group compared with the vehicle (Supplementary Figure S2A). Similar to *Bcl9* pharmacological inhibition, the *Bcl9* depletion also affected the interaction of macrophages and T cells, where CSF1R-CSF1 and CCL4-CCR5 were significantly regulated (Supplementary Figure S2B). Our data showed that targeting Wnt pathway through hsBCL9_CT_-24 treatment very likely inhibit macrophages and enhance T-cell activity (Supplementary Figures S2C,D).

### Immunohistochemical Analysis of Tumor-Associated Macrophages Markers of CSF1R and Cxcl14 Genes in Colorectal Cancer

We re-analyzed the human CRC immunohistochemical pictures downloaded from the public database the human protein atlas (https://www.proteinatlas.org/). The results showed that the *CSF1R* as the M2 marker gene was highly expressed in CRC. The survival curve data indicated that the prognosis in the high *GSF1R* expression group was poorer than that of the low expression group (Supplementary Figures S3A–C). The *CSCL14* gene as a marker of M1 was moderately or weakly expressed in CRC. The survival curve data showed better prognosis in group with high expression of *CXCL14* than in low *CXCL14* expressing groups (Supplementary Figures S3D–F).

## Discussion

Tumor microenvironment is an important factor affecting tumor treatment and prognosis. Because of its dramatic impact on tumor occurrence and development, as well as heterogeneity, it has attracted growing interest in oncology, especially in consideration of new targets for tumor therapy. TAMs are one of the major cellular components of most cancer microenvironments; more than 50% of tumor-infiltrating cells are TAMs. In this study, we demonstrated that the cellular landscape and transcription differences of TAMs after BCL9 suppression affected cancer and immune surveillance.

TAMs are an extremely heterogeneous population in CRC, and their characteristics are strongly dependent on the TME. Patients with infiltrating TAMs in CRC LMs have poorer prognosis than patients without TAM infiltration in LMs (Cortese et al., 2019). TAMs play an immunosuppressive role by secreting cytokines and chemokines and also can activate the expression of immune regulatory proteins in T cells (Cortese et al., 2019). We found that *Bcl9* suppression changed macrophages polarization into the subtype M1 and M2. Macrophage polarization is increasingly understood as an essential pathogenetic factor in inflammatory and neoplastic diseases.

Macrophages are abundant in CRC (Yahaya et al., 2019), where they are recruited from and where they may mediate metastasis-promoting communication. Their interactions with cancer cells as well as their crosstalk with the TME have not yet been elucidated. CRC is the most common cancer in the world, so fighting this disease presents an enormous therapeutic challenge. Traditional treatment (i.e., surgery, radiotherapy, and chemotherapy combined with targeted drugs) has advanced the treatment for early-stage CRC over the years. Metastases, however, are the leading cause of death and remain poorly understood (Tauriello et al., 2017). Recent reports have demonstrated that Wnt signaling pathway activation induces primary resistance to immunotherapy. Macrophages can interact with the Wnt signaling pathway (Malsin et al., 2019).

After undergoing cluster analysis (Figure 2 and Figure 3), pseudotime analysis (Figure 4), signal pathway enrichment analysis (Figure 4), and transcription factor analysis (Supplementary Figure S1), we found that *Bcl9* deprivation in TME inhibited the differentiation of M0 to M2, and inhibited the inflammatory response caused by TAMs.

The downstream genes of the circadian rhythms-regulated transcription factor Per1 may have important contributions to these biological processes. The circadian clock in mammals is controlled by three cell-autonomous feedback loops. The first loop includes two activators (*CLOCK* and *BMAL1*) and four repressors (*PER1*, *PER2*, *CRY1*, and *CRY2*). PER1 is downregulated in many types of cancers in the Cancer Genome Atlas. Heterozygous deletion of *PER1* also has been observed in cancer (Wu et al., 2019). Circadian disruption was found to change tumor-immune microenvironment, favoring tumor cell proliferation (Aiello et al., 2020). In profile 2 of cardiac dysfunction, the Wnt/beta-catenin pathway was identified with activation (Lecarpentier et al., 2014). Our finding of negative regulation of *Per1* in TIME of colon tumor provided evidence for BCL9-driven Wnt signaling role in circadian disruption.

Delineation of immune cells and their dynamic balance are a growing research field [61]. Their existence and role in tumors, however, are rarely reported. We found TAM-derived NK-like non-functioning cells in our samples, and this type of cells showed significant differences in the BCL9-deprived cell population. Changes in markers on the surface of these cells and their cell biological functions may significantly affect the TME, which in turn affects the growth and metastasis of tumors and the prognosis in patients. To evaluate the impact of this dynamic balance on tumor prognosis, we proposed TAM-to-NK score, which can evaluate the homeostasis of TAM and NK-like non-functioning cell populations in tumors at the level of gene expression. As shown in Figure 8, TAM-to-NK score can indeed predict the prognosis of patients in some specific cancers. In the future, further experimental research on NK-like non-functioning cells in the tumor microenvironment is very necessary. This will focus on using specific antibodies to stain newly discovered markers or perform mRNA *in situ* hybridization.

Although targeted TME has great potential for tumor treatment, the treatment of advanced metastatic CRCs remains challenging; therefore, exploring new target molecules and therapeutic strategies is of paramount importance. M2-like TAMs are believed to induce tumor-supporting, angiogenic, and immunosuppressive effects and also may induce failure of immunotherapy (Mantovani et al., 2017). Defining the biology of macrophages present in the CRC-specific environment could allow the introduction of innovative diagnostic and therapeutic strategies. TAMs affect many aspects of tumor biology, including stem cells, metabolism, angiogenesis, invasion, and metastasis virtually. The research progress of Wnt/β-catenin signaling pathway mechanisms accelerates the discovery of new therapeutic methods targeting the Wnt/β-catenin pathway in CRC. Although most drugs are still in early stages of research, there is hope that drugs to cure intractable CRC are on the horizon. Our findings have highlighted the potential of targeting the BCL9-driven Wnt signaling pathway in TMAs in cancer immunotherapy.

## Novelty and Impact

Changes in the BCL9-mediated signaling pathway caused changes in the balance of cell populations in the tumor microenvironment to affect the prognosis of cancer patients.

## Data Availability

The datasets presented in this study can be found in online repositories. The names of the repository/repositories and accession number(s) can be found in the article/Supplementary Material.

## Supplementary Material

Supplementary Figure S1 GSVA analysis of M0, M1 and M2. Transcription factor analysis for M0 **(A)**; M1 **(B)**, and M2 **(C)** TAM cells classified by perturbation of BCL9 or not, MsigDB C3 (TFT: transcription factor targets) is used.

Supplementary Figure S2 Cell-cell Interaction between macrophage cells and T cells in the colorectal microenvironment. **(A)** Interaction analysis between CD8 + T and CT26 cells in NT-shRNA group. **(B)** Interaction analysis between CD8 + T and CT26 cells in hsBCL9CT-24 treated group. **(C)** Circos plots of all of the putative ligand–receptor interactions in NT-shRNA and Bcl9-shRNA groups. The solid line represents the interaction of Bcl9-shRNA group, and the dotted line represents the interaction of the NT-shRNA group. **(D)** Circos plots of all of the putative ligand–receptor interactions in vehicle and hsBCL9CT-24 treated groups. The color of the line represents the average expression of the ligand receptor pair in the pair of cells, and the thickness of the line represents the significance of the ligand receptor enrichment in the pair of cells. The solid line represents the interaction of the hsbcl9CT-24 group, and the dotted line represents the interaction of the vehicle group.

Supplementary Figure S3 Immunohistochemistry analysis of TAM marker of CSF1R and Cxcl14 gene in colorectal cancer. Immunohistochemistry was carried in human colorectal cancer. **(A–C)** CSF1R express in colorectal cancer and survival analysis. **(D,E)** Cxcl14 express and survival analysis in human colorectal cancer. The data were derived from https://www.proteinatlas.org/ENSG00000145824-CXCL14/pathology/liver+cancer#imid_7374796

## Abbreviations

CRC, colorectal cancer; DFS, disease-free survival; GSEA, Gene Set Enrichment Analysis; GSVA, Gene Set Variation Analysis; HCC, human hepatocellular carcinoma; HPLC, high-performance liquid chromatography; HR, hazard ratio; hsbcl9, hsBCL9CT-24; KD, Bcl9-shRNA; Maxstat, maximally selected statistical status; MMR, mismatch repair; MS, mass spectrometry; NT, Non-targeting shRNA; OS, overall survival; PCA, Principal component analysis; Per1, Period Circadian Regulator 1; RRID, Research Resource Identifier; TAMs, Tumor-associated macrophages; TFT, transcription factor targets; TME, tumor microenvironment; t-SNE, t-distributed stochastic neighbor embedding; UMAP, Uniform Manifold Approximation and Projection; UMI, unique molecular identifier; Veh, vehicle

## References

[B1] Aiello I. Fedele M. L. M. Román F. Marpegan L. Caldart C. Chiesa J. J. (2020). Circadian Disruption Promotes Tumor-Immune Microenvironment Remodeling Favoring Tumor Cell Proliferation. Sci. Adv. 6, eaaz4530. 10.1126/sciadv.aaz4530 33055171 PMC7556830

[B2] Bergenfelz C. Medrek C. Ekström E. Jirström K. Janols H. Wullt M. (2012). Wnt5a Induces a Tolerogenic Phenotype of Macrophages in Sepsis and Breast Cancer Patients. J. Immunol. 188, 5448–5458. 10.4049/jimmunol.1103378 22547701

[B3] Blumenthal A. Ehlers S. Lauber J. Buer J. Lange C. Goldmann T. (2006). The Wingless Homolog WNT5A and its Receptor Frizzled-5 Regulate Inflammatory Responses of Human Mononuclear Cells Induced by Microbial Stimulation. Blood 108, 965–973. 10.1182/blood-2005-12-5046 16601243

[B4] Cavnar M. J. Turcotte S. Katz S. C. Kuk D. Gönen M. Shia J. (2017). Tumor-Associated Macrophage Infiltration in Colorectal Cancer Liver Metastases Is Associated with Better Outcome. Ann. Surg. Oncol. 24, 1835–1842. 10.1245/s10434-017-5812-8 28213791 PMC5505689

[B5] Chen Q. Ye W. Jian Tan W. Mei Yong K. S. Liu M. Qi Tan S. (2015). Delineation of Natural Killer Cell Differentiation from Myeloid Progenitors in Human. Sci. Rep. 5, 15118. 10.1038/srep15118 26456148 PMC4600975

[B6] Cortese N. Soldani C. Franceschini B. Barbagallo M. Marchesi F. Torzilli G. (2019). Macrophages in Colorectal Cancer Liver Metastases. Cancers (Basel) 11, 633. 10.3390/cancers11050633 PMC6562719 31067629

[B7] Coussens L. M. Werb Z. (2002). Inflammation and Cancer. Nature 420, 860–867. 10.1038/nature01322 12490959 PMC2803035

[B8] Edin S. Wikberg M. L. Rutegård J. Oldenborg P. A. Palmqvist R. (2013). Phenotypic Skewing of Macrophages *In Vitro* by Secreted Factors from Colorectal Cancer Cells. PLoS One 8, e74982. 10.1371/journal.pone.0074982 24058644 PMC3776729

[B9] Fang Y. Kang Y. Zou H. Cheng X. Xie T. Shi L. (2018). β-Elemene Attenuates Macrophage Activation and Proinflammatory Factor Production via Crosstalk with Wnt/β-Catenin Signaling Pathway. Fitoterapia 124, 92–102. 10.1016/j.fitote.2017.10.015 29066299

[B10] Feng M. Jin J. Q. Xia L. Xiao T. Mei S. Wang X. (2019). Pharmacological Inhibition of β-catenin/BCL9 Interaction Overcomes Resistance to Immune Checkpoint Blockades by Modulating Treg Cells. Sci. Adv. 5, eaau5240. 10.1126/sciadv.aau5240 31086813 PMC6506245

[B11] Feng Q. Chang W. Mao Y. He G. Zheng P. Tang W. (2019). Tumor-associated Macrophages as Prognostic and Predictive Biomarkers for Postoperative Adjuvant Chemotherapy in Patients with Stage II Colon Cancer. Clin. Cancer Res. 25, 3896–3907. 10.1158/1078-0432.CCR-18-2076 30988081

[B12] Feng Y. Liang Y. Ren J. Dai C. (2018). Canonical Wnt Signaling Promotes Macrophage Proliferation during Kidney Fibrosis. Kidney Dis. (Basel) 4, 95–103. 10.1159/000488984 29998124 PMC6029229

[B13] Feng Y. Ren J. Gui Y. Wei W. Shu B. Lu Q. (2018). Wnt/β-Catenin-Promoted Macrophage Alternative Activation Contributes to Kidney Fibrosis. J. Am. Soc. Nephrol. 29, 182–193. 10.1681/ASN.2017040391 29021383 PMC5748914

[B14] Forssell J. Oberg A. Henriksson M. L. Stenling R. Jung A. Palmqvist R. (2007). High Macrophage Infiltration along the Tumor Front Correlates with Improved Survival in colon Cancer. Clin. Cancer Res. 13, 1472–1479. 10.1158/1078-0432.CCR-06-2073 17332291

[B15] Hao N. B. Lü M. H. Fan Y. H. Cao Y. L. Zhang Z. R. Yang S. M. (2012). Macrophages in Tumor Microenvironments and the Progression of Tumors. Clin. Dev. Immunol. 2012, 948098. 10.1155/2012/948098 22778768 PMC3385963

[B16] Isidro R. A. Appleyard C. B. (2016). Colonic Macrophage Polarization in Homeostasis, Inflammation, and Cancer. Am. J. Physiol. Gastrointest. Liver Physiol. 311, G59–G73. 10.1152/ajpgi.00123.2016 27229123 PMC4967174

[B17] Jati S. Kundu S. Chakraborty A. Mahata S. K. Nizet V. Sen M. (2018). Wnt5A Signaling Promotes Defense against Bacterial Pathogens by Activating a Host Autophagy Circuit. Front. Immunol. 9, 679. 10.3389/fimmu.2018.00679 29686674 PMC5900007

[B18] Lan J. Sun L. Xu F. Liu L. Hu F. Song D. (2019). M2 Macrophage-Derived Exosomes Promote Cell Migration and Invasion in Colon Cancer. Cancer Res. 79, 146–158. 10.1158/0008-5472.CAN-18-0014 30401711

[B19] Lecarpentier Y. Claes V. Duthoit G. Hébert J. L. (2014). Circadian Rhythms, Wnt/beta-Catenin Pathway and PPAR Alpha/gamma Profiles in Diseases with Primary or Secondary Cardiac Dysfunction. Front. Physiol. 5, 429. 10.3389/fphys.2014.00429 25414671 PMC4220097

[B20] Lee Y. S. Song S. J. Hong H. K. Oh B. Y. Lee W. Y. Cho Y. B. (2020). The FBW7-MCL-1 axis Is Key in M1 and M2 Macrophage-Related colon Cancer Cell Progression: Validating the Immunotherapeutic Value of Targeting PI3Kγ. Exp. Mol. Med. 52, 815–831. 10.1038/s12276-020-0436-7 32444799 PMC7272616

[B21] Li J. Li L. Li Y. Long Y. Zhao Q. Ouyang Y. (2020). Tumor-associated Macrophage Infiltration and Prognosis in Colorectal Cancer: Systematic Review and Meta-Analysis. Int. J. Colorectal Dis. 35, 1203–1210. 10.1007/s00384-020-03593-z 32303831

[B22] Liberzon A. Subramanian A. Pinchback R. Thorvaldsdóttir H. Tamayo P. Mesirov J. P. (2011). Molecular Signatures Database (MSigDB) 3.0. Bioinformatics 27, 1739–1740. 10.1093/bioinformatics/btr260 21546393 PMC3106198

[B23] Luput L. Licarete E. Sesarman A. Patras L. Alupei M. C. Banciu M. (2017). Tumor-associated Macrophages Favor C26 Murine colon Carcinoma Cell Proliferation in an Oxidative Stress-dependent Manner. Oncol. Rep. 37, 2472–2480. 10.3892/or.2017.5466 28260079

[B24] Macciò A. Gramignano G. Cherchi M. C. Tanca L. Melis L. Madeddu C. (2020). Role of M1-Polarized Tumor-Associated Macrophages in the Prognosis of Advanced Ovarian Cancer Patients. Sci. Rep. 10, 6096. 10.1038/s41598-020-63276-1 32269279 PMC7142107

[B25] Maiti G. Naskar D. Sen M. (2012). The Wingless Homolog Wnt5a Stimulates Phagocytosis but Not Bacterial Killing. Proc. Natl. Acad. Sci. U S A. 109, 16600–16605. 10.1073/pnas.1207789109 23012420 PMC3478623

[B26] Malsin E. S. Kim S. Lam A. P. Gottardi C. J. (2019). Macrophages as a Source and Recipient of Wnt Signals. Front. Immunol. 10, 1813. 10.3389/fimmu.2019.01813 31417574 PMC6685136

[B27] Mantovani A. Marchesi F. Malesci A. Laghi L. Allavena P. (2017). Tumour-associated Macrophages as Treatment Targets in Oncology. Nat. Rev. Clin. Oncol. 14, 399–416. 10.1038/nrclinonc.2016.217 28117416 PMC5480600

[B28] Mao J. Wang D. Wang Z. Tian W. Li X. Duan J. (2016). Combretastatin A-1 Phosphate, a Microtubule Inhibitor, Acts on Both Hepatocellular Carcinoma Cells and Tumor-Associated Macrophages by Inhibiting the Wnt/β-Catenin Pathway. Cancer Lett. 380, 134–143. 10.1016/j.canlet.2016.06.020 27349166

[B29] Naskar D. Maiti G. Chakraborty A. Roy A. Chattopadhyay D. Sen M. (2014). Wnt5a-Rac1-NF-κB Homeostatic Circuitry Sustains Innate Immune Functions in Macrophages. J. Immunol. 192, 4386–4397. 10.4049/jimmunol.1302817 24706725

[B30] Ong S. M. Tan Y. C. Beretta O. Jiang D. Yeap W. H. Tai J. J. (2012). Macrophages in Human Colorectal Cancer Are Pro-inflammatory and Prime T Cells towards an Anti-tumour Type-1 Inflammatory Response. Eur. J. Immunol. 42, 89–100. 10.1002/eji.201141825 22009685

[B31] Ono D. Honma S. Nakajima Y. Kuroda S. Enoki R. Honma K. I. (2017). Dissociation of Per1 and Bmal1 Circadian Rhythms in the Suprachiasmatic Nucleus in Parallel with Behavioral Outputs. Proc. Natl. Acad. Sci. U S A. 114, E3699–E3708. 10.1073/pnas.1613374114 28416676 PMC5422828

[B32] Pereira C. Schaer D. J. Bachli E. B. Kurrer M. O. Schoedon G. (2008). Wnt5A/CaMKII Signaling Contributes to the Inflammatory Response of Macrophages and Is a Target for the Antiinflammatory Action of Activated Protein C and Interleukin-10. Arterioscler Thromb. Vasc. Biol. 28, 504–510. 10.1161/ATVBAHA.107.157438 18174455

[B33] Raghavan S. Mehta P. Xie Y. Lei Y. L. Mehta G. (2019). Ovarian Cancer Stem Cells and Macrophages Reciprocally Interact through the WNT Pathway to Promote Pro-tumoral and Malignant Phenotypes in 3D Engineered Microenvironments. J. Immunother. Cancer 7, 190. 10.1186/s40425-019-0666-1 31324218 PMC6642605

[B34] Ruffell B. Affara N. I. Coussens L. M. (2012). Differential Macrophage Programming in the Tumor Microenvironment. Trends Immunol. 33, 119–126. 10.1016/j.it.2011.12.001 22277903 PMC3294003

[B35] Sarode P. Zheng X. Giotopoulou G. A. Weigert A. Kuenne C. Günther S. (2020). Reprogramming of Tumor-Associated Macrophages by Targeting β-catenin/FOSL2/ARID5A Signaling: A Potential Treatment of Lung Cancer. Sci. Adv. 6, eaaz6105. 10.1126/sciadv.aaz6105 32548260 PMC7274802

[B36] Sawa-Wejksza K. Dudek A. Lemieszek M. Kaławaj K. Kandefer-Szerszeń M. (2018). Colon Cancer-Derived Conditioned Medium Induces Differentiation of THP-1 Monocytes into a Mixed Population of M1/M2 Cells. Tumour Biol. 40, 1010428318797880. 10.1177/1010428318797880 30183516

[B37] Shalhoub J. Falck-Hansen M. A. Davies A. H. Monaco C. (2011). Innate Immunity and Monocyte-Macrophage Activation in Atherosclerosis. J. Inflamm. (Lond) 8, 9. 10.1186/1476-9255-8-9 21526997 PMC3094203

[B38] Subramanian A. Tamayo P. Mootha V. K. Mukherjee S. Ebert B. L. Gillette M. A. (2005). Gene Set Enrichment Analysis: a Knowledge-Based Approach for Interpreting Genome-wide Expression Profiles. Proc. Natl. Acad. Sci. U S A. 102, 15545–15550. 10.1073/pnas.0506580102 16199517 PMC1239896

[B39] Tauriello D. V. Calon A. Lonardo E. Batlle E. (2017). Determinants of Metastatic Competency in Colorectal Cancer. Mol. Oncol. 11, 97–119. 10.1002/1878-0261.12018 28085225 PMC5423222

[B40] Tian X. Wu Y. Yang Y. Wang J. Niu M. Gao S. (2020). Long Noncoding RNA LINC00662 Promotes M2 Macrophage Polarization and Hepatocellular Carcinoma Progression via Activating Wnt/β-Catenin Signaling. Mol. Oncol. 14, 462–483. 10.1002/1878-0261.12606 31785055 PMC6998656

[B41] Tran M. T. N. Hamada M. Jeon H. Shiraishi R. Asano K. Hattori M. (2017). MafB Is a Critical Regulator of Complement Component C1q. Nat. Commun. 8, 1700. 10.1038/s41467-017-01711-0 29167450 PMC5700178

[B42] Vinnakota K. Zhang Y. Selvanesan B. C. Topi G. Salim T. Sand-Dejmek J. (2017). M2-like Macrophages Induce colon Cancer Cell Invasion via Matrix Metalloproteinases. J. Cel Physiol 232, 3468–3480. 10.1002/jcp.25808 28098359

[B43] Wallace J. Lutgen V. Avasarala S. St Croix B. Winn R. A. Al-Harthi L. (2018). Wnt7a Induces a Unique Phenotype of Monocyte-Derived Macrophages with Lower Phagocytic Capacity and Differential Expression of Pro- and Anti-inflammatory Cytokines. Immunology 153, 203–213. 10.1111/imm.12830 28872671 PMC5765374

[B44] Waniczek D. Lorenc Z. Śnietura M. Wesecki M. Kopec A. Muc-Wierzgoń M. (2017). Tumor-Associated Macrophages and Regulatory T Cells Infiltration and the Clinical Outcome in Colorectal Cancer. Arch. Immunol. Ther. Exp. (Warsz) 65, 445–454. 10.1007/s00005-017-0463-9 28343267 PMC5602054

[B45] Wei Z. Feng M. Wu Z. Shen S. Zhu D. (2020). Bcl9 Depletion Modulates Endothelial Cell in Tumor Immune Microenvironment in Colorectal Cancer Tumor. Front. Oncol. 10, 603702. 10.3389/fonc.2020.603702 33552975 PMC7856347

[B46] Wu Y. Tao B. Zhang T. Fan Y. Mao R. (2019). Pan-Cancer Analysis Reveals Disrupted Circadian Clock Associates with T Cell Exhaustion. Front. Immunol. 10, 2451. 10.3389/fimmu.2019.02451 31708917 PMC6821711

[B47] Yahaya M. A. F. Lila M. A. M. Ismail S. Zainol M. Afizan N. A. R. N. M. (2019). Tumour-Associated Macrophages (TAMs) in Colon Cancer and How to Reeducate Them. J. Immunol. Res. 2019, 2368249. 10.1155/2019/2368249 30931335 PMC6410439

[B48] Yang Y. Ye Y. C. Chen Y. Zhao J. L. Gao C. C. Han H. (2018). Crosstalk between Hepatic Tumor Cells and Macrophages via Wnt/β-Catenin Signaling Promotes M2-like Macrophage Polarization and Reinforces Tumor Malignant Behaviors. Cell Death Dis 9, 793. 10.1038/s41419-018-0818-0 30022048 PMC6052107

[B49] Yuan A. Hsiao Y. J. Chen H. Y. Chen H. W. Ho C. C. Chen Y. Y. (2015). Opposite Effects of M1 and M2 Macrophage Subtypes on Lung Cancer Progression. Sci. Rep. 5, 14273. 10.1038/srep14273 26399191 PMC4585843

[B50] Yuan C. Yang D. Ma J. Yang J. Xue J. Song F. (2020). Modulation of Wnt/β-Catenin Signaling in IL-17A-mediated Macrophage Polarization of RAW264.7 Cells. Braz. J. Med. Biol. Res. 53, e9488. 10.1590/1414-431X20209488 32578719 PMC7307890

[B51] Zhou Y. Zhou B. Pache L. Chang M. Khodabakhshi A. H. Tanaseichuk O. (2019). Metascape Provides a Biologist-Oriented Resource for the Analysis of Systems-Level Datasets. Nat. Commun. 10, 1523. 10.1038/s41467-019-09234-6 30944313 PMC6447622

[B52] Zhu Z. Yin S. Wu K. Lee A. Liu Y. Li H. (2018). Downregulation of Sfrp5 in Insulin Resistant Rats Promotes Macrophage-Mediated Pulmonary Inflammation through Activation of Wnt5a/JNK1 Signaling. Biochem. Biophys. Res. Commun. 505, 498–504. 10.1016/j.bbrc.2018.09.070 30268495

