# Supplementary figures and images for: Inhibition of BCL9 Modulates the Cellular Landscape of Tumor-Associated Macrophages in the Tumor Immune Microenvironment of Colorectal Cancer
Source: Front Pharmacol. 2021 Sep 10;12:713331. doi: 10.3389/fphar.2021.713331 (PMC8461101; doi:10.3389/fphar.2021.713331)

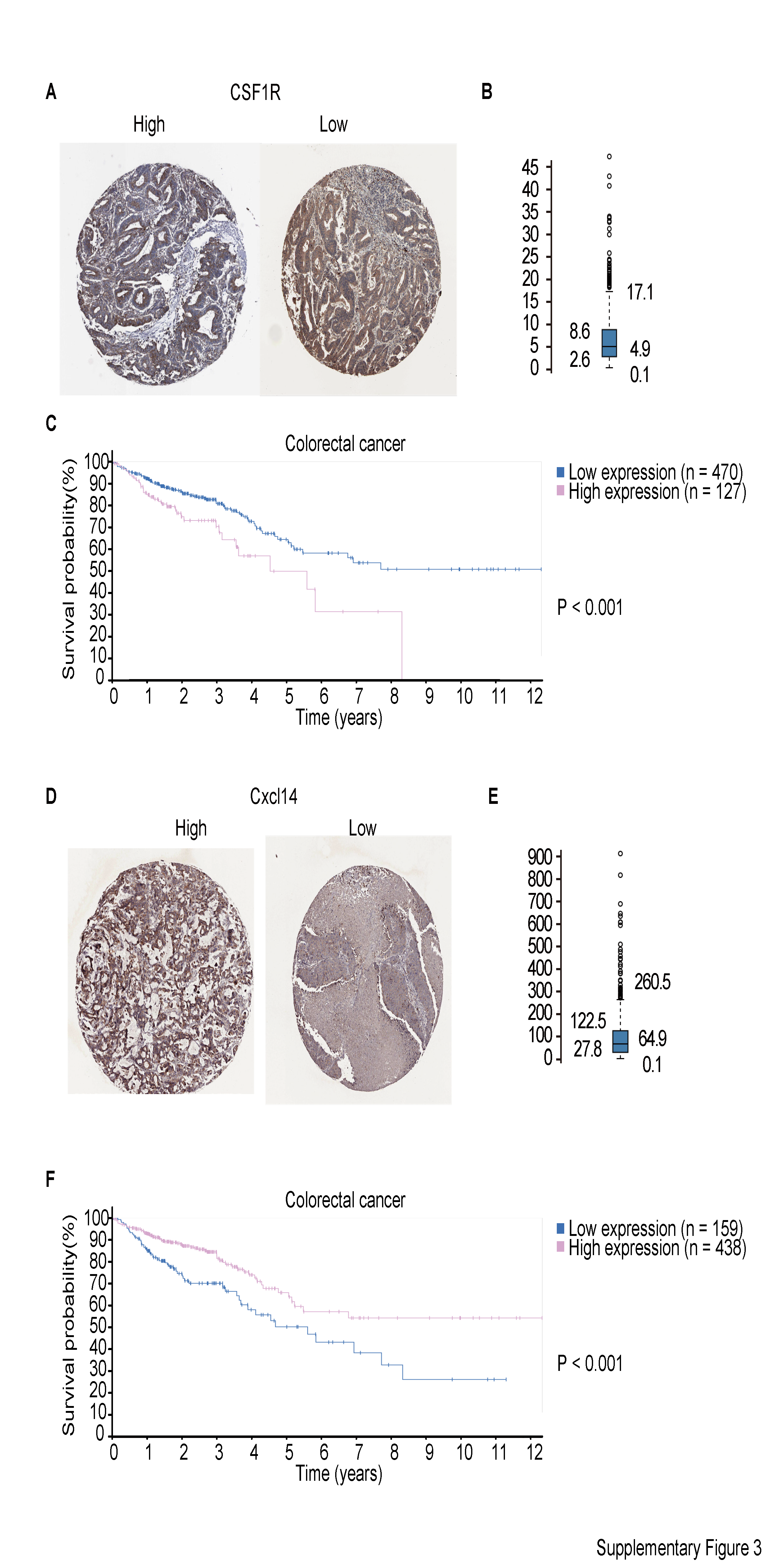

Supplement: Supplementary file 1 [file Image3.TIFF]

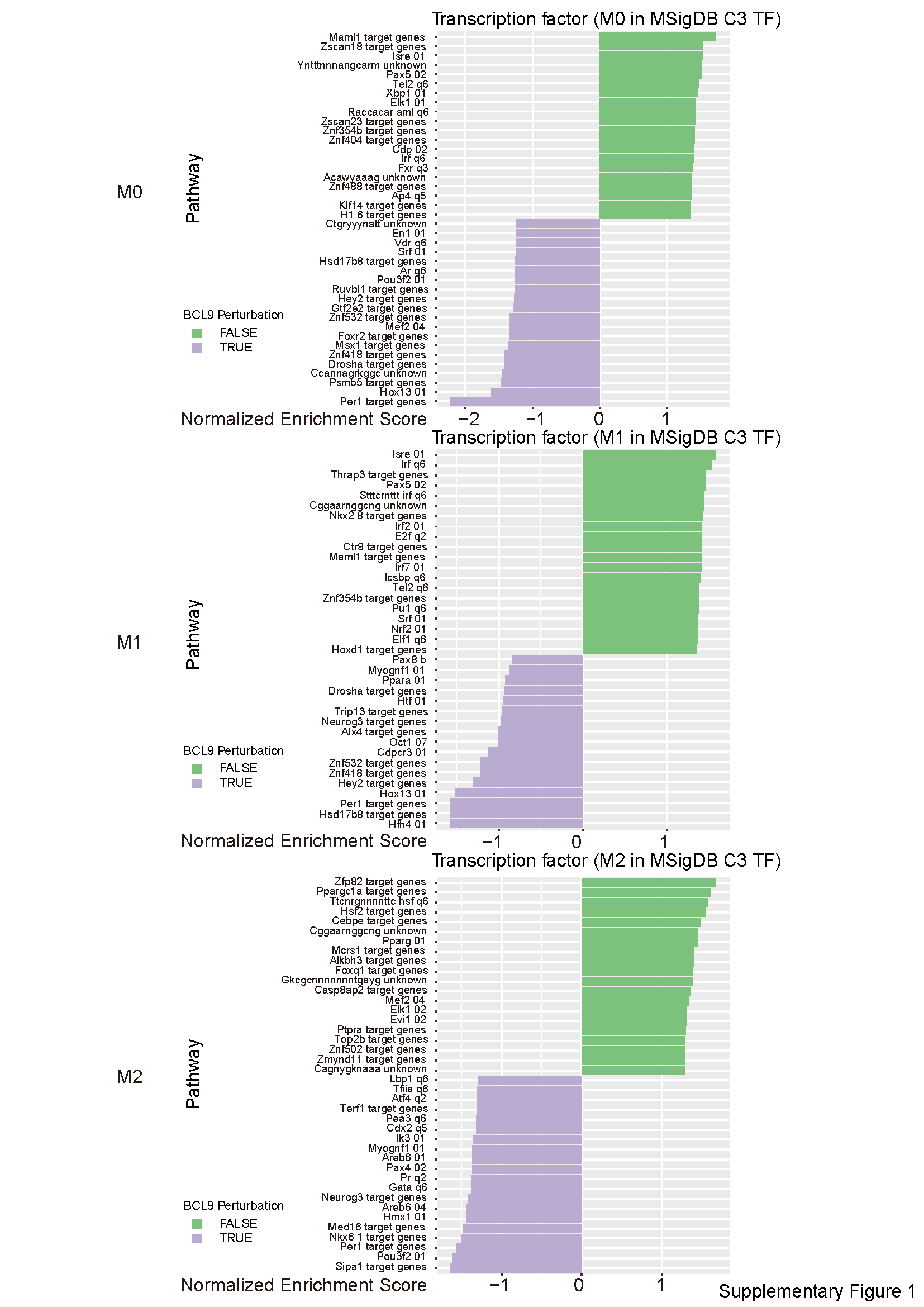

Supplement: Supplementary file 2 [file Image1.TIFF]

## Tumor microenvironment monocyte-macrophage differentiation

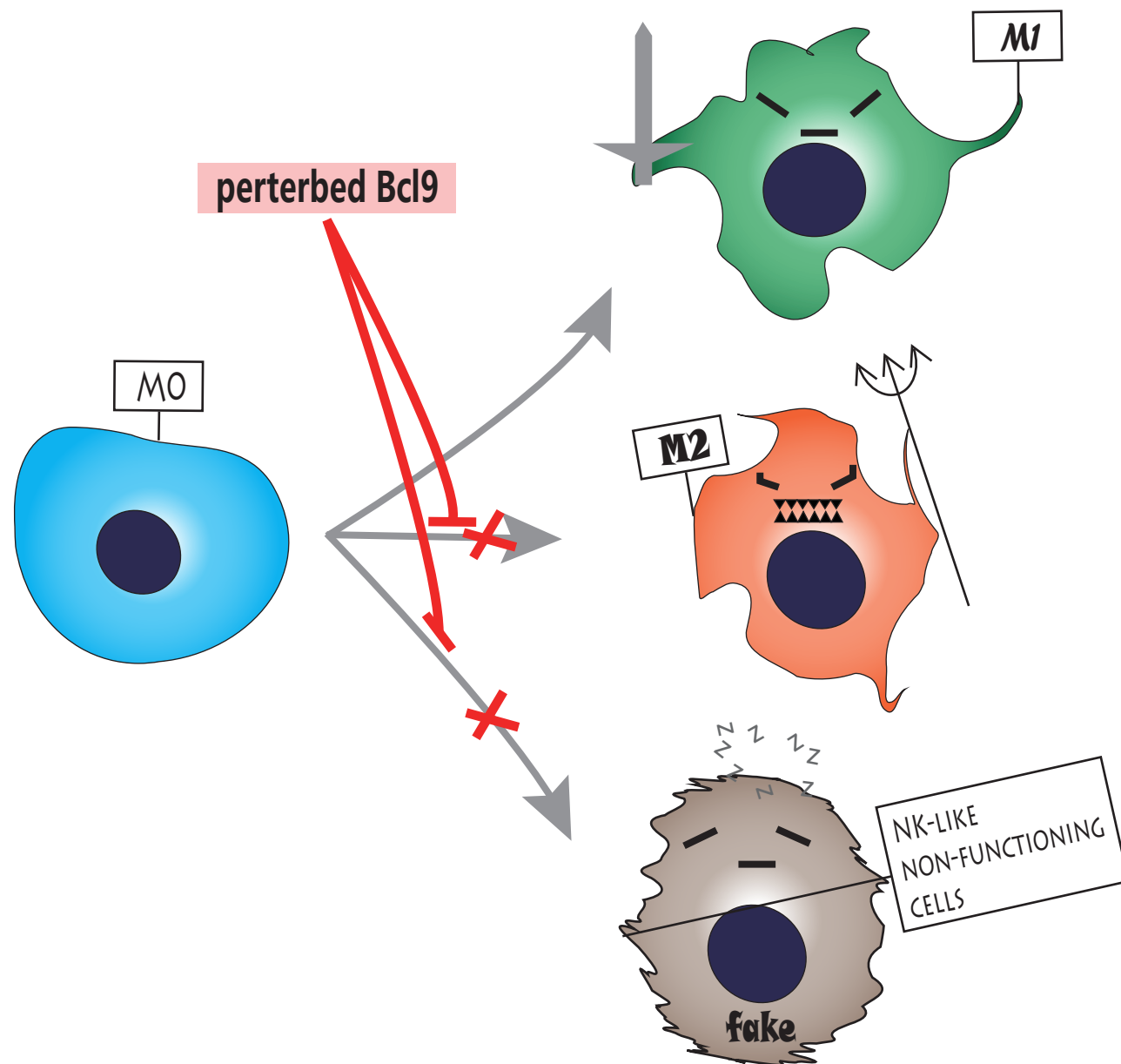

Supplement: Supplementary file 3 [file Image4.pdf]

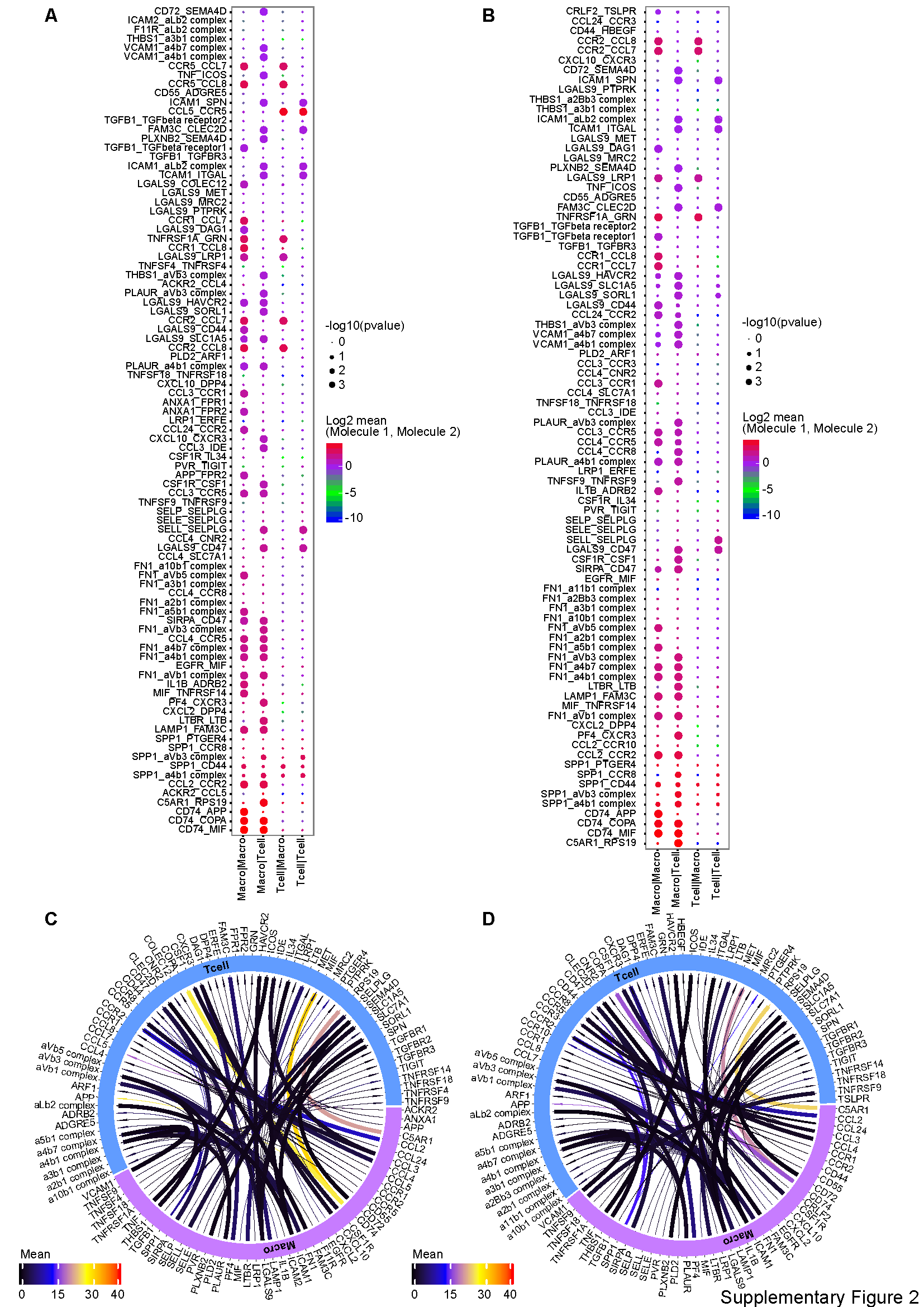

Supplement: Supplementary file 4 [file Image2.TIFF]
